# Inguinal Hernia Recurrence in Adults in Romania: A Five-Year Nationwide Analysis of Surgical Practice and Health System Disparities

**DOI:** 10.3390/medicina62020391

**Published:** 2026-02-17

**Authors:** Anca Tigora, Dragos Garofil, Mihai Zurzu, Vlad Paic, Mircea Bratucu, Florian Popa, Valeriu Surlin, Sandu Ramboiu, Daniela Marinescu, Victor Strambu, Petru Radu

**Affiliations:** 1 General Surgery Department, Carol Davila Nephrology Hospital, 020021 Bucharest, Romania; anca.tigora@drd.umfcd.ro (A.T.); mihai.zurzu@drd.umfcd.ro (M.Z.); vlad.paic@drd.umfcd.ro (V.P.); mircea.bratucu@umfcd.ro (M.B.); florian.popa@umfcd.ro (F.P.); petru.radu@umfcd.ro (P.R.); 2 Tenth Department of Surgery, University of Medicine and Pharmacy “Carol Davila”, 050474 Bucharest, Romania; 3 Sixth Department of Surgery, University of Medicine and Pharmacy of Craiova, Craiova Emergency Clinical 7 Hospital, 200642 Craiova, Romania; valeriu.surlin@umfcv.ro (V.S.); sandu.ramboiu@umfcv.ro (S.R.); daniela.marinescu@umfcv.ro (D.M.); 4 Academy of Romanian Scientists, Ilfov Streets, nr 3, 050044 Bucharest, Romania

**Keywords:** inguinal hernia recurrence, public/private hospital, laparoscopic repair

## Abstract

*Introduction*: Recurrent inguinal hernia remains a clinically relevant outcome that is difficult to quantify in the absence of national prospective registries. In Romania, structural differences between public and private hospitals may further influence recurrence-related care, access to minimally invasive surgery, and resource utilization. This study aimed to assess recurrence patterns after inguinal hernia repair at a national level, with emphasis on reinterventions, patient-related risk factors, and health system disparities. *Methods*: A nationwide retrospective cohort study was conducted using administrative DRG data from the Romanian National Health Insurance House. All adult patients undergoing inguinal hernia repair in 2019 were identified and followed for five years (2019–2023). Reintervention was used as a proxy for recurrence. Surgical approach, hospital sector, length of stay, reimbursement, patient migration, geographic distribution, and comorbidities were analyzed using descriptive statistics and multivariable logistic regression to explore factors associated with laparoscopic approach and reintervention. *Results*: Among the 18,185 patients who underwent inguinal hernia repair in 2019, reintervention rates during follow-up ranged from 0.58% to 4.88%, a variability that reflects inherent limitations of administrative coding. Most reinterventions occurred in the year of the index surgery, suggesting early technical failure. Public hospitals managed the majority of cases and disproportionately absorbed recurrent and clinically complex patients. Access to laparoscopic repair was uneven and concentrated in large academic centers. Length of hospital stay declined gradually in public hospitals but remained consistently shorter in private institutions, reflecting differences in patient selection and care pathways. Reimbursement by The National Health Insurance House was similar for open and laparoscopic procedures. *Conclusions*: Recurrent inguinal hernia care in Romania is shaped by system-level disparities extending beyond surgical technique. Further progress requires reimbursement reform, establishment of a national hernia registry, and expansion of laparoscopic training to ensure equitable access to high-quality hernia care.

## 1. Introduction

Inguinal hernia repair represents the most frequently performed procedure in general surgery with approximately 20 million interventions carried out worldwide each year [1]. This condition accounts for approximately 75% of all abdominal wall hernias and affects an estimated 27% of men and 3% of women over their lifetime [2]. Surgical intervention remains the definitive management for groin hernia, and although numerous repair techniques have been described in the literature over the decades, the introduction of mesh-based approaches has been associated with a marked reduction in recurrence rates [3].

Management of recurrent inguinal hernia continues to pose a considerable challenge for surgeons, owing to the technical complexity of reintervention and its association with elevated postoperative morbidity and a persistent risk of further relapse [4,5,6]. For inguinal hernia, reported recurrence rates vary according to surgical approach and follow-up methodology. Registry-based studies and large clinical series indicate that recurrence or reintervention rates after inguinal hernia repair generally range between approximately 1% and 10% [7,8,9]. Specifically, recurrence rates following laparoscopic inguinal hernia repair over the past two decades have been reported to range from approximately 1% to 7.9% [7,10,11].

Prospective documentation of groin hernia repairs was initiated with the objective of establishing dedicated national registries, thereby enabling systematic evaluation of major developments in clinical practice, including the adoption of mesh-based repairs, laparo-endoscopic approaches and the wider implementation of day-case surgery [12]. Although several hernia registries have subsequently been developed in Europe and North America, nationwide coverage that includes all adult patients and surgical units remains uncommon [13]. In Europe, the Swedish Hernia Register, introduced in 1992, and the Danish Hernia Database, established in 1997 with participation mandated by public decree, are the only hernia registries that provide nationwide coverage [14].

International studies suggest that the uptake of minimally invasive procedures is strongly shaped by national healthcare policies and the availability of medical infrastructure. Recent Brazilian data indicate that, among more than 700,000 inguinal hernia procedures performed within the Unified Health System between 2017 and 2022, open repair was used in 99.2% of cases [15]. Similarly, a large population-based study from Spain involving over 260,000 patients reported that 94.3% of repairs were carried out using an open approach [16]. Findings from a review conducted by the Americas Hernia Society Quality Collaborative further demonstrated a substantial reliance on open surgery among North American surgeons, with a reported rate of 42% [17]. In Italy, registry data reveal a gradual increase in the adoption of minimally invasive techniques; however, their utilization remains below internationally recommended levels [18]. In contrast, Denmark has widely embraced laparoscopic hernia repair as the standard approach, with national registry data demonstrating that this strategy is associated with improved recurrence rates and more favorable postoperative outcomes [19].

Romania lacks a national prospective hernia database, which limits the systematic evaluation of surgical practices within a healthcare system divided between public and private hospitals that adopt different approaches to the management of inguinal hernia patients. Private institutions, supported by more flexible financing mechanisms and direct patient contributions, tend to implement new technologies more rapidly, whereas public hospitals often operate under financial and logistical constraints. Nevertheless, public hospitals remain the principal providers of care for the majority of the population, particularly for patients unable to access private services. Building on our previous research describing nationwide trends in groin hernia repair in Romania [20], the present study examines the management of recurrent inguinal hernia over a five-year period (2019–2023), comparing surgical activity in public and private hospitals, evaluating the adoption of minimally invasive techniques, and identifying trends in laparoscopic repair, with the aim of highlighting institutional differences and potential areas for improvement in surgical care.

## 2. Material and Methods

We performed a retrospective, nationwide analysis encompassing all patients (18,185 cases) who underwent inguinal hernia repair in Romania during the year 2019. The study utilized anonymized DRG data supplied by the National Institute of Health Services Management (INMSS), encompassing the complete national network of Romanian healthcare institutions authorized to perform groin hernia surgery. In total, 272 hospitals were included in the analysis, of which 231 were public and 41 private. By identifying this national patient population and following it over a five-year period, we sought not only to determine the long-term recurrence rate, but also to capture the broader trajectory of therapeutic approaches and surgical practices related to inguinal hernia within both the public and private sectors of the Romanian healthcare system. This framework allowed for a comprehensive assessment of how national management patterns have evolved for this common surgical condition.

We identified all adult patients (≥18 years) who underwent surgical management for inguinal hernia in Romania in 2019 using ICD-10 surgical procedure codes, specifically: laparoscopic repair of unilateral femoral hernia (J12701), laparoscopic repair of bilateral femoral hernia (J12702), open repair of unilateral femoral hernia (J12703), open repair of bilateral femoral hernia (J12704), laparoscopic repair of unilateral inguinal hernia (J12601), laparoscopic repair of bilateral inguinal hernia (J12602), open repair of unilateral inguinal hernia (J12603), and open repair of bilateral inguinal hernia (J12604). Although femoral hernia repair procedure codes were included at the index surgery identification stage to ensure comprehensive capture of groin hernia repairs within the national DRG framework, recurrence tracking during follow-up was restricted to ICD-10 codes specific to recurrent inguinal hernia. Femoral hernia recurrences are inconsistently coded in administrative datasets and were therefore not included as recurrence events. Femoral repairs represented a small minority of index procedures and did not contribute to the recurrence numerator.

During the subsequent five-year follow-up period, potential recurrences were identified by querying hospitalizations assigned diagnostic codes for recurrent unilateral inguinal hernia (K40.31, K40.41, K40.91) or recurrent bilateral inguinal hernia (K40.01, K40.11, K40.21), using the unique anonymized patient identifier to ensure patient-level linkage. In addition, we verified repeated admissions for groin hernia repair, thereby ensuring a comprehensive assessment of recurrence events and subsequent surgical interventions. Recurrence was not directly observable in the administrative DRG dataset. Therefore, two complementary proxies were used to estimate recurrence after inguinal hernia repair during a fixed five-year follow-up period (2019–2023), both using the same denominator of 18,185 patients operated in 2019. First, a minimum recurrence estimate was derived from hospitalizations explicitly coded with an ICD-10 diagnosis of recurrent inguinal hernia. Second, a maximum estimate was derived from any subsequent reintervention for groin hernia during follow-up, irrespective of diagnostic coding

Independent variables included institutional characteristics (hospital type), demographic factors (age and sex), clinical parameters (recurrence status and hernia laterality), the presence of comorbidities, as well as hospitalization duration and costs. The dependent variable was the surgical technique employed, categorized as either open repair or laparoscopic repair.

To assess differences in hospitalization duration, preoperative and postoperative length of stay, we conducted comparative statistical analyses between laparoscopic and open surgery, as well as between public and private hospitals. Because the distributions of these variables deviated from normality (Shapiro–Wilk test, *p* < 0.05), non-parametric methods were applied. The Mann–Whitney U test was used to compare surgical approaches and hospital types for each hospitalization parameter, while the Kruskal–Wallis test was employed to examine the combined effect of surgical technique and hospital category. Statistical significance was defined as *p* < 0.05. Length of stay variables are reported as mean values; measures of dispersion were not available in the administrative dataset.

To account for potential confounding related to patient characteristics and hospital sector, multivariable logistic regression analyses were performed. Two separate models were constructed. The first model evaluated factors associated with the likelihood of undergoing laparoscopic inguinal hernia repair at the index hospitalization. The second model assessed factors associated with the need for reintervention during the five-year follow-up period, using reintervention as a proxy for recurrence. Covariates were selected a priori based on clinical relevance and data availability and included age, sex, hospital sector (public vs. private), and comorbidities identified as relevant in univariable analyses (diabetes mellitus, benign prostatic hyperplasia, and obesity). Surgical approach and year of surgery were included in the models where applicable. Results are reported as odds ratios (ORs) with 95% confidence intervals. Model fit was assessed using standard diagnostic procedures. Due to the structure of the DRG database, hernia laterality and hospital-level clustering could not be accounted for in the regression models. Propensity scorebased methods were considered; however, their use was limited by the absence of essential clinical variables in the administrative dataset, including hernia type, defect size, surgical technique, mesh characteristics, and surgeon volume. In the absence of these key confounders, propensity-based matching was unlikely to achieve adequate covariate balance and was therefore not applied.

Because individual-level length of stay (LOS) distributions deviated from normality, non-parametric tests were used for the primary comparisons between surgical approaches and hospital sectors. For exploratory comparisons between the pandemic (2020–2021) and post-pandemic (2022–2023) periods, analyses were performed on aggregated annual mean LOS values within predefined subgroups. In this context, Welch’s *t*-test was applied as a robust parametric method allowing for unequal variances. Effect sizes, expressed as mean differences with 95% confidence intervals, were prioritized for interpretation.

Ethical approval was granted by the Ethics Committee of the Carol Davila Clinical Nephrology Hospital (approval no. 111/22 December 2025), which confirmed that the use of fully anonymized data was in accordance with ethical standards and that informed consent was not required.

## 3. Results

We limited the analysis to patients whose initial hernia repair was performed in 2019 in order to ensure an adequate follow-up interval and to improve the reliability of recurrence rate estimates. In that year, a total of 18,444 patients underwent hernia surgery; however, 259 individuals coded as having recurrent hernia were excluded, as these cases corresponded to procedures performed in a prior period. Among the remaining 18,185 patients, 888 required at least one subsequent surgical intervention during follow-up, and 29 patients underwent three or more reinterventions. While the latter group can be reasonably interpreted as representing true recurrences, cases involving only two hospitalizations are more difficult to classify with certainty. *Patients undergoing three or more groin hernia–related surgical admissions during follow-up were considered highly suggestive of true recurrence, as postoperative complications such as hematoma, infection, or seroma are rarely managed surgically more than once and are typically coded under different diagnostic categories In addition, repeat admissions for postoperative complications generally occur within a short postoperative window, whereas multiple reinterventions observed in this cohort were frequently separated by longer intervals, supporting the interpretation of recurrence rather than complication management.*

Only 107 of the 18,185 patients had a documented diagnosis of recurrent hernia at the time of a second or third intervention, corresponding to a minimum estimated recurrence rate of 0.58%. This figure should be regarded as a lower bound, as multiple inconsistencies and errors in the coding of recurrent hernia diagnoses were identified. This limitation is further illustrated by the fact that, among the 29 patients who underwent three interventions, only six had a recurrence diagnosis recorded at the third procedure, underscoring substantial underreporting within administrative data.

An additional methodological challenge arises from the structure of the diagnosis-related group (DRG) coding system, which does not differentiate between right- and left-sided inguinal hernias. Consequently, patients undergoing two separate operations may represent either true recurrences or staged repairs of bilateral disease, introducing further uncertainty in the interpretation of reintervention rates. If recurrence were estimated solely on the basis of reoperated patients, the maximum national recurrence rate would be calculated as 4.88% (888 of 18,185 patients). However, this estimate is likely inflated due to the inability to distinguish recurrences from bilateral procedures. Taken together, these considerations indicate that the true recurrence rate most plausibly lies between the minimum value derived from diagnostic coding and the upper bound suggested by reintervention frequency (0.58–4.88%). The reported recurrence range of 0.58–4.88% represents cumulative five-year proportions derived using two distinct methodological approaches applied to the same national cohort of 18,185 patients. The lower bound (0.58%) corresponds to patients with an explicit ICD-10 diagnosis code for recurrent inguinal hernia recorded at reintervention, whereas the upper bound (4.88%) reflects the proportion of patients who underwent at least one subsequent groin hernia reintervention during follow-up, irrespective of recurrence coding. The upper estimate should be interpreted as a maximal boundary, as the administrative dataset does not allow differentiation between true recurrence and staged bilateral repair or other groin reinterventions.

Therefore, the database included 18,185 inguinal hernia cases operated in 2019, of which 15,619 were treated using an open surgical approach and 2566 underwent laparoscopic repair. The patient population was predominantly male, accounting for 87.86% of cases, with a mean age of 60 years (±16). Most procedures were performed in public hospitals (17,388 cases), with only a small fraction occurring in private institutions (797 cases) and a substantial number of surgeries took place in university-affiliated medical centers (10,786 cases). It is important to note that these numbers include only procedures reimbursed through the National Health Insurance House (NHIH), as privately financed surgeries performed outside the NHIH reporting system are not captured in the dataset. In multivariable analysis adjusting for age, sex, hospital sector, and selected comorbidities, hospital sector and patient age remained independently associated with the likelihood of laparoscopic repair.

**Figure 1:**
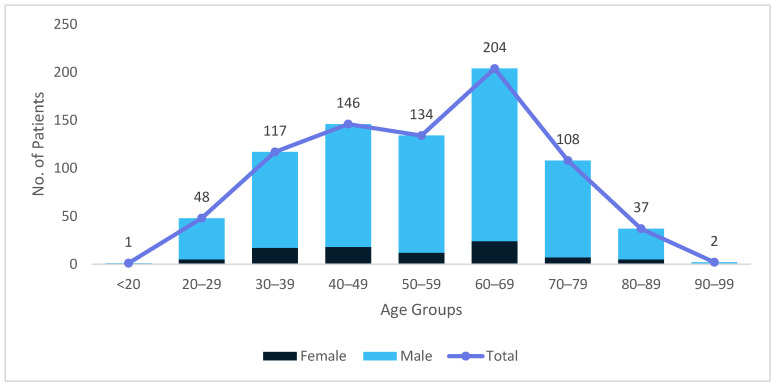
Private sector patient distribution according to age and sex.

Figure 1 presents the age and sex specific distribution of patients treated for inguinal hernia in the private sector, illustrating a characteristic epidemiological pattern. The number of cases increases progressively from early adulthood, reaching its peak in the 60–69 age group, after which it declines gradually in older age categories. Males consistently predominate across all age groups, reflecting the well-established sex disparity associated with inguinal hernia. Very few cases occur at the extremes of age (<20 and ≥90 years), while individuals aged 40–69 represent the majority of interventions. This distribution underscores the concentration of private-sector surgical activity among older adults and highlights the demographic profile of patients seeking care for this condition in private medical settings.

**Figure 2:**
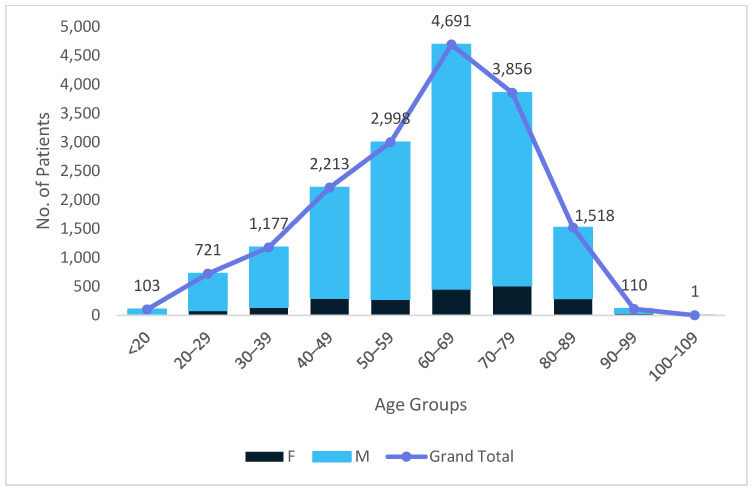
Public Sector Patient Distribution According to Age and Sex.

Figure 2 depicts the distribution of patients undergoing inguinal hernia repair in public hospitals across demographic categories, demonstrating a pattern that mirrors the general trend observed in the private sector, yet with substantially greater magnitude across all age groups. As in private facilities, case numbers rise progressively from young adulthood and peak in the 60–69 age group; however, public hospitals treat markedly higher volumes of patients in every age category. The predominance of male patients is again evident across all age groups, reflecting the established epidemiology of inguinal hernia, yet the absolute number of male cases in public institutions far exceeds that in private settings, particularly between ages 50 and 79. Public hospitals also exhibit a wider distribution at the extremes of age, managing more patients < 20 years of age and very elderly (≥90 years) patients than private institutions, likely reflecting differences in access, referral pathways, and case-mix complexity. Overall, while both sectors display similar demographic trends, the public system manages a substantially larger and more heterogeneous patient population, underscoring its central role in the national provision of surgical care for inguinal hernia.

**Figure 3:**
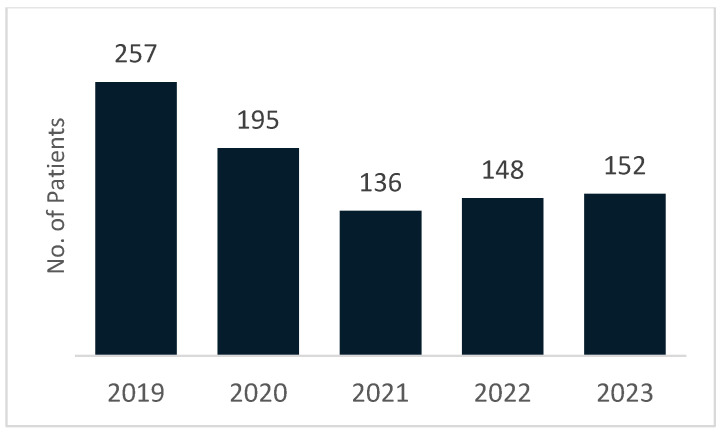
Annual Distribution of Recurrence Interventions.

Across the five-year follow-up period (2019–2023), a total of 888 patients originally operated for inguinal hernia in 2019 required reintervention for recurrence. Figure 3 illustrates the annual distribution of these reinterventions, showing a marked decline from 2019 (257 cases) to 2021 (136 cases), followed by a gradual increase in 2022 (148 cases) and 2023 (152 cases). Notably, the highest number of recurrence-related reinterventions occurred in 2019, the same year as the index surgery, indicating that a substantial proportion of recurrences manifested early, a pattern consistent with early postoperative failure described in the literature. The subsequent decrease likely reflects reduced access to elective surgery during the COVID-19 pandemic, while the gradual rise in later years suggests a progressive return to normal surgical activity. Overall, the temporal pattern demonstrates that recurrence-related reinterventions were not uniformly distributed, but were shaped by both clinical factors and system-level disruptions within the healthcare system.

**Figure 4:**
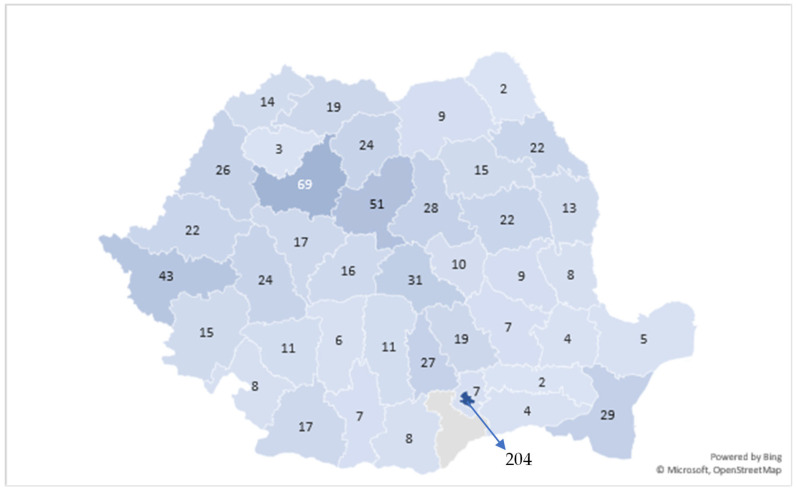
Geographic Distribution of Reinterventions for Recurrent Inguinal Hernia in Romania.

Figure 4 illustrates the geographic distribution of the 888 reinterventions for recurrent inguinal hernia across Romania’s counties, revealing substantial regional variability in recurrence-related surgical activity. The highest concentrations are observed in major urban and academic centers, with Bucharest registering the largest number of cases (204), followed by counties such as Cluj (69 cases), Iași (51 cases), and Timiș (43 cases). These areas correspond to major academic medical centers and tertiary referral hospitals, which likely receive a significant number of complex or recurrent cases from surrounding regions.

In contrast, several counties report markedly lower numbers of reinterventions—often fewer than 10 cases—reflecting smaller populations, reduced surgical capacity, or limited referral patterns. The uneven spatial distribution suggests that recurrence-related surgical care is concentrated in counties with advanced surgical infrastructure and high-volume centers, while sparsely populated or less medically resourced regions contribute proportionally fewer cases. Overall, the map highlights the central role of large metropolitan and university-affiliated counties in managing recurrent inguinal hernia, while also illustrating the nationwide presence of recurrence requiring surgical correction.

We analyzed the migration of patients between the private and public sectors at the time of reintervention for recurrent inguinal hernia, as illustrated in Table 1, which reveals a clear asymmetry. Among patients initially operated in private hospitals, one quarter (6 out of 24) migrated to the public system for their recurrence surgery, whereas patient flow in the opposite direction was minimal: of the 864 patients whose primary intervention occurred in the public sector, only 20 individuals (2.3%) shifted to private hospitals for reintervention, while the overwhelming majority (844 patients) continued to be treated within the public system.

These patterns indicate that patient migration predominantly occurs from private to public hospitals rather than the reverse. This likely reflects differences in cost coverage, accessibility, and the organization of surgical care, with public hospitals serving as the primary destination for most recurrence-related procedures—even for patients initially treated in the private sector.

We evaluated sex-specific differences in recurrence following inguinal hernia repair and identified a clear disparity between male and female patients. Although women represented a smaller proportion of the overall surgical population, their recurrence rate was 3.81%, compared with 5.03% in men. This pattern indicates that male patients not only account for the majority of primary hernia repairs but also experience a higher likelihood of requiring reintervention. The findings are consistent with the well-documented sex differences in hernia pathophysiology, where anatomical and biomechanical factors contribute to a greater predisposition to recurrence in men.

**Figure 5:**
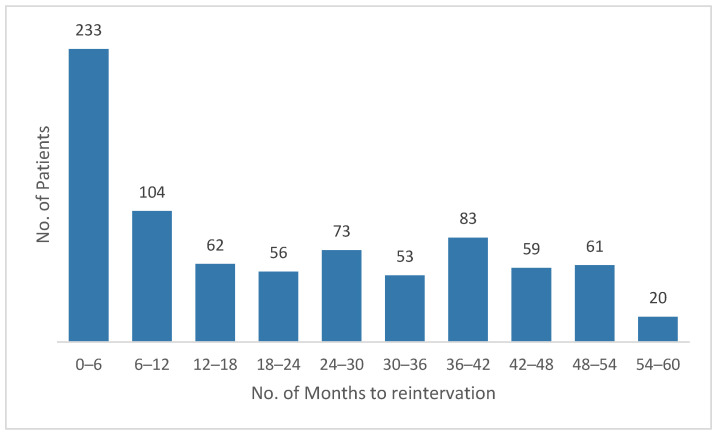
Distribution of Recurrence Interventions in Male Patients by Months Since Index Surgery.

**Figure 6:**
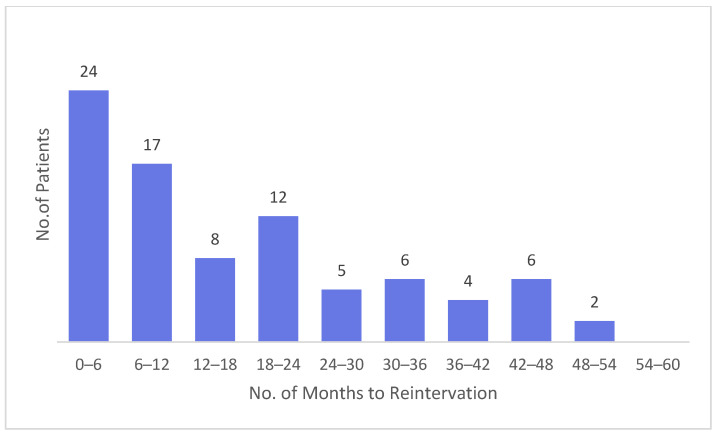
Distribution of Recurrence Interventions in Female Patients by Months Since Index Surgery.

A comparative analysis of recurrence timing in males and females demonstrates a consistent pattern across both sexes, with the majority of reinterventions occurring early after the index surgery (Figure 5 and Figure 6). In men, recurrences are heavily concentrated within the first 6 months (233 cases), followed by a sharp decline in subsequent intervals, whereas in women, although the absolute numbers are lower, the same early clustering is observed, with 24 cases in the first 6 months and 17 cases in the 6–12-month period. After the first postoperative year, both groups show a more dispersed distribution of reinterventions, with smaller counts spread across the remaining follow-up intervals. Males exhibit a modest secondary rise around 36–42 months, while female cases remain relatively low and stable throughout the later time frames. Overall, the comparative pattern indicates that early recurrences dominate in both sexes—suggesting shared mechanisms of early postoperative failure—while late recurrences are less frequent and more evenly distributed over time, with men showing a wider temporal spread due to their much larger case volume.

**Figure 7:**
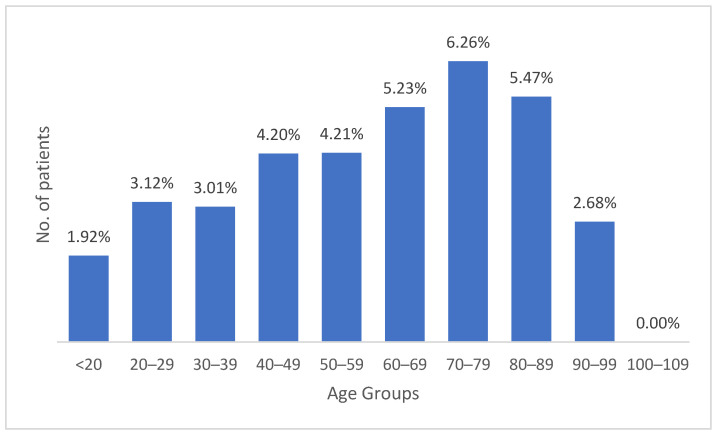
Age-Related Variation in Inguinal Hernia Recurrence Rates.

Figure 7 presents the recurrence rate across age groups, revealing a clear age-dependent pattern in the likelihood of hernia recurrence. Recurrence rates are lowest in patients under 20 years of age (1.92%) and rise progressively through adulthood, reaching a peak of 6.26% in the 70–79-year age group before declining to 2.68% among patients aged 90–99 years. This upward trend indicates that advancing age is associated with a higher risk of postoperative hernia failure, likely reflecting age-related changes in connective tissue quality, increased comorbidity burden, and reduced physiological resilience. A Chi-square test of independence confirmed a statistically significant association between age group and recurrence rate (χ^2^ = 40.08, df = 9, *p* < 0.001), demonstrating that older patients had a markedly greater likelihood of experiencing recurrence. The lower-than-expected recurrence rate in individuals over 90 years may reflect selection bias, as very elderly or frail patients with recurrent hernia are less frequently selected for reintervention and thus are underrepresented in surgical databases.

We examined in detail the relationship between common comorbidities and the likelihood of requiring reintervention for recurrent inguinal hernia, and the results reveal several clinically meaningful patterns (Table 2). Although hypertension was the most prevalent comorbidity in the cohort (6218 patients), its recurrence rate did not differ significantly from that of non-hypertensive patients (*p* = 0.4097), indicating that blood pressure status alone is unlikely to influence postoperative failure. Obesity and anemia showed recurrence tendencies that were numerically higher compared with the general cohort, yet neither reached statistical significance (*p* = 0.0920 and *p* = 0.0870, respectively), suggesting potential but unconfirmed associations that might become significant in larger samples or in subgroups with severe disease.

Among the comorbidities evaluated, diabetes mellitus and benign prostatic hyperplasia were more frequently observed among patients undergoing reintervention for recurrent inguinal hernia. These associations are consistent with prior reports suggesting that metabolic dysfunction and conditions associated with chronic straining may be overrepresented in patients experiencing postoperative failure. However, the observed relationships are based on unadjusted comparisons derived from administrative data and should be interpreted cautiously, as they may reflect differences in age, overall disease burden, or care pathways rather than independent effects. The proposed biological mechanisms should therefore be regarded as plausible explanatory hypotheses rather than definitive causal pathways.

Cardiac comorbidities—heart failure, ischemic heart disease, and atrial fibrillation showed no significant association with recurrence (*p* > 0.44 for all). Despite their clinical relevance, these conditions do not appear to influence the mechanical or biological factors that directly contribute to hernia recurrence. Similarly, chronic kidney disease and urinary tract infections were not associated with increased recurrence risk.

Overall, this analysis suggests that metabolic dysfunction (diabetes) and urological conditions associated with chronic straining (BPH) represent the most relevant comorbid predictors of recurrence in this population, while major cardiovascular diseases do not appear to alter recurrence risk. These findings highlight the importance of optimizing metabolic control and managing lower urinary tract symptoms prior to or following hernia repair in order to reduce the risk of postoperative recurrence.

**Figure 8:**
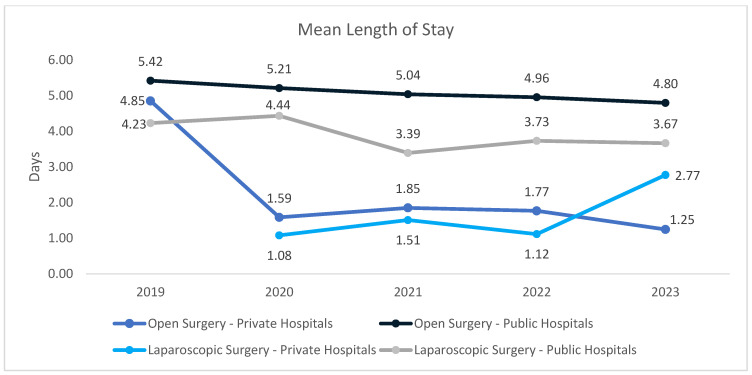
Mean Length of Hospital Stay for Recurrent Inguinal Hernia Repair According to Surgical Approach and Hospital Sector.

We analyzed the mean LOS for patients undergoing inguinal hernia repair in public and private hospitals, comparing open and laparoscopic approaches to evaluate the impact of surgical technique and hospital setting on inpatient resource utilization, while also identifying substantial sector- and approach-specific differences with distinct temporal patterns confirmed by regression and comparative analyses (Figure 8).

In public hospitals, open inguinal hernia repair demonstrated a clear and progressive reduction in LOS, decreasing from 5.42 days in 2019 to 4.80 days in 2023. Linear regression analysis confirmed a statistically significant downward trend over time (slope −0.149 days/year, *p* = 0.0013), indicating gradual improvements in perioperative management and discharge practices. Laparoscopic repair in public hospitals was associated with shorter LOS compared with open surgery; however, the temporal trend was modest and did not reach statistical significance (slope −0.183 days/year, *p* = 0.214), with LOS values ranging from 4.23 days in 2019 to 3.67 days in 2023.

In private hospitals, LOS was markedly shorter for both surgical approaches. Open surgery exhibited an abrupt reduction from 4.85 days in 2019 to approximately 1.6–1.9 days between 2020 and 2022, reaching 1.25 days in 2023. This pattern reflects a stepwise structural change rather than a gradual temporal trend, and accordingly, the overall linear trend was not statistically significant (*p* = 0.138). Laparoscopic repair in private hospitals showed the lowest LOS overall but displayed greater variability, ranging from 1.08 to 2.77 days, with no significant linear trend over time (*p* = 0.236).

To assess the potential impact of the COVID-19 pandemic, LOS during the pandemic period (2020–2021) was compared with the post-pandemic interval (2022–2023) using two-sample Welch *t*-tests. Across all subgroups, observed differences in mean LOS between pandemic and post-pandemic periods were small, with overlapping 95% confidence intervals, indicating limited clinical relevance. No statistically significant differences were identified within any surgical or institutional category (all *p* > 0.17), suggesting that the pronounced reduction observed between 2019 and 2020 in private open surgery likely reflects a sustained organizational or policy-driven change rather than a transient pandemic-related effect.

Overall, these findings indicate that while public hospitals have achieved gradual improvements in LOS over time, private hospitals exhibit consistently shorter hospital stays, particularly following open surgery, highlighting structural differences in care pathways and resource utilization between the two healthcare sectors.

The analysis of reimbursement costs for reinterventions following inguinal hernia repair, based on average payments from the National Health Insurance House (CNAS), demonstrates notable variations according to surgical approach and hospital sector (Table 3). Overall, the mean reimbursement across all reinterventions was 2568.89 RON. When stratified by technique, laparoscopic reinterventions were associated with higher average costs compared with open (classic) procedures (2992.67 RON vs. 2508.35 RON, respectively).

Within the open surgery group, reimbursement levels were relatively similar between hospital sectors, although slightly higher in public hospitals (2513.48 RON) compared with private institutions (2353.90 RON). In contrast, laparoscopic reinterventions exhibited a more pronounced sectoral disparity, with public hospitals reporting the highest average reimbursement overall (3063.51 RON), exceeding both laparoscopic procedures in private hospitals (2458.63 RON) and all open reinterventions.

These findings suggest that laparoscopic reinterventions impose a greater financial burden on the healthcare system, particularly within the public sector, likely reflecting increased procedural complexity, longer operative times, higher equipment and consumable costs, and differences in reimbursement structures. The lower costs observed in private hospitals, especially for laparoscopic procedures, may be related to more streamlined care pathways, different case selection, or variations in contractual reimbursement mechanisms. Collectively, these data highlight the economic implications of surgical approach and institutional setting in the management of recurrent inguinal hernia and underscore the importance of cost-conscious adoption of minimally invasive techniques within publicly funded healthcare systems.

## 4. Discussion

Our analysis in Romania reveals an estimated recurrence rate for patients undergoing inguinal hernia repair ranging between 0.58% and 4.88%. The peak in recurrence-related reinterventions was observed in 2019, coinciding with the year of the primary operation, a pattern consistent with early technical failure. However, in the context of administrative DRG data, early reinterventions cannot be attributed exclusively to technical failure. Alternative explanations include staged repair of bilateral disease, miscoding of recurrent hernia, or reinterventions for early postoperative complications such as hematoma or infection, which cannot be reliably distinguished from true recurrence in the absence of operative detail. The subsequent reduction in reintervention volume during 2020–2021 likely reflects reduced access to elective surgery during the COVID-19 pandemic, followed by a gradual normalization of surgical activity in 2022–2023. This interpretation is supported by national-level data demonstrating a substantial reduction in elective groin hernia surgery during the COVID-19 pandemic. A Romanian nationwide DRG-based analysis reported a 44.45% decrease in groin hernia repair volume in 2020 compared with 2019, with surgical activity remaining 29.72% below pre-pandemic levels in 2021. The most pronounced decline occurred during the first lockdown, when the number of procedures performed in April 2020 dropped by approximately 94% [21]. International registry-based studies consistently report lower recurrence or reintervention rates following inguinal hernia repair compared with those observed in administrative datasets, although methodological differences limit direct comparisons. Data from established national hernia registries in Denmark, Sweden, the United Kingdom, The United States of America, and Spain generally indicate recurrence or reintervention rates in the low single-digit range, particularly in high-volume centers and standardized care pathways. Variability across countries reflects differences in case-mix, surgical technique, registry completeness, and outcome definitions, underscoring the importance of cautious interpretation when comparing international recurrence estimates [9,22,23,24,25,26,27]. Collectively, these data underscore the wide international variability in reported recurrence rates and emphasize the critical influence of methodology, follow-up duration, and data source on recurrence estimates.

Direct comparison of recurrence rates across countries is limited by substantial heterogeneity in follow-up duration, outcome definitions, and data sources. Several cited studies report recurrence or reintervention rates at different time horizons (1-year, 5-year, or 10-year), which are not directly comparable. In many registry-based and administrative datasets, reintervention is used as a proxy for recurrence, whereas clinical follow-up studies may capture both operated and non-operated recurrences, further contributing to methodological heterogeneity. Where time-matched five-year recurrence estimates were unavailable, international comparisons in the present study are intended to be descriptive rather than quantitative and are interpreted within this methodological context.

Importantly, comparisons between public and private hospitals are strongly influenced by differences in patient case-mix, referral pathways, and institutional organization, and should therefore be interpreted as descriptive rather than causal; within this context, the present analysis is intended to characterize national patterns of care and system-level disparities rather than to attribute differences in outcomes to hospital type.

The present analysis suggests that recurrence after inguinal hernia repair is driven by selective comorbid conditions that directly affect tissue integrity or impose sustained mechanical stress on the repair. The significant associations observed for diabetes mellitus and benign prostatic hyperplasia are consistent with established biological and biomechanical mechanisms, including impaired collagen remodeling and wound healing in diabetic patients and chronic increases in intra-abdominal pressure in those with lower urinary tract obstruction [28]. The molecular alterations described by Radu et al. further support this interpretation by linking hernia failure to dysregulated extracellular matrix turnover [29]. In contrast, the absence of an association with major cardiovascular comorbidities indicates that systemic disease alone does not necessarily translate into increased recurrence risk. Collectively, these observations highlight the importance of targeted perioperative optimization of metabolic status and urological conditions to mitigate recurrence risk.

Beyond recurrence estimation, the present analysis identifies substantial differences between public and private hospitals in Romania across several key dimensions, including case volume and complexity, referral and migration patterns, geographic access to care, patient selection mechanisms, and institutional capacity for organizational adaptation.

First, there is a marked imbalance in case volume and case-mix complexity between the two sectors. In Romania public hospitals treat a broader (95.6%) and more heterogeneous patient population, including a higher proportion of elderly patients, individuals at the extremes of age, and patients with comorbidities associated with recurrence risk, such as diabetes mellitus and benign prostatic hyperplasia. This uneven distribution of clinical complexity likely contributes to the longer hospitalization durations and higher reintervention burden observed in the public sector and must be considered when comparing outcomes between hospital types. Similar patterns have been described specifically for recurrent inguinal hernia repair in other countries. Population-based and registry studies indicate that recurrent cases are disproportionately managed in public or high-volume referral centers, where patients tend to be older and present with a higher burden of comorbidities and technical complexity.

Registry-based data consistently indicate that recurrent inguinal hernia repair is associated with greater operative complexity, increased resource utilization, and higher postoperative morbidity, and is preferentially managed within public-sector or high-volume centers. Analyses from Denmark and Sweden show longer hospital stays and higher morbidity among patients undergoing surgery for recurrent hernia, reflecting both case complexity and referral patterns toward public institutions [22,23,24]. Similar trends have been reported in the United Kingdom and the United States, where recurrent hernia reinterventions are disproportionately concentrated in public or safety-net hospitals and are associated with older age and increased comorbidity burden [25,26]. Collectively, these data support the notion that recurrent inguinal hernia represents a distinct clinical entity that is preferentially treated in public-sector or high-volume centers, and that comparisons between hospital types must account for this systematic imbalance.

Moreover, the public healthcare system functions as a national safety net for recurrent and complex cases, as demonstrated by the asymmetric migration of patients at the time of reintervention. In Romania among patients initially operated on in private hospitals, 25% subsequently receive treatment in public hospitals for recurrence, whereas migration in the opposite direction remains minimal (2.3%). This pattern suggests that recurrence-related surgical care is predominantly managed by public institutions, regardless of the sector in which the primary repair is performed. Hemberg et al. conducted a register-based study in Sweden that directly compared outcomes of groin hernia repair between public and private healthcare settings, demonstrating that sector-specific organizational structures influence patient pathways and surgical outcomes [30]. Danish registry work likewise shows that hospital type/volume relates to recurrence-related reintervention after laparoscopic inguinal hernia repair, consistent with the idea that more complex cases and failures may be managed within more specialized or higher-capacity units [31].

In our country access to laparoscopic inguinal hernia repair is unevenly distributed, reflecting both sectoral and geographic disparities. Although laparoscopic techniques show a gradual increase over the study period, their use remains largely concentrated in major urban and academic centers. This distribution suggests that access to minimally invasive surgery is influenced not only by hospital ownership but also by regional differences in surgeon training, equipment availability, and institutional priorities. Similar geographic and institutional disparities in access to laparoscopic inguinal hernia repair have been reported internationally. Population-based analyses from Italy demonstrate that minimally invasive hernia surgery is predominantly performed in high-volume urban and university-affiliated centers, while smaller regional hospitals exhibit markedly lower adoption rates, largely attributable to differences in surgeon training and availability of laparoscopic equipment [18]. Comparable findings are reported in Spain, where the use of laparoscopic repair is strongly associated with treatment in large tertiary hospitals and metropolitan areas, reflecting regional inequalities in infrastructure and expertise [16].

Intersectoral differences in length of stay appear largely driven by patient selection and institutional care models. In public hospitals, length of stay decreased gradually over time, with open repair consistently associated with longer hospitalization than laparoscopic surgery. By contrast, private hospitals demonstrated persistently shorter hospital stays for both techniques, particularly for open repair, reflecting short-stay care pathways. These patterns remained stable across the study period, including during the COVID-19 pandemic, suggesting that observed differences are attributable to structural and organizational factors rather than transient system disruptions. Consistent with these findings, international evidence indicates that laparoscopic inguinal hernia repair is generally associated with shorter hospital stays compared with open techniques [32]. Direct observations documented in diverse healthcare systems comparing LOS for recurrent inguinal hernia repair between public and private hospitals is limited.

Despite the well-established clinical benefits of laparoscopic inguinal hernia repair, the current Romanian reimbursement framework does not adequately account for the greater resource requirements of minimally invasive reinterventions, particularly in the public sector. The National Health Insurance House (NHIH) applies a largely uniform reimbursement scheme to both open and laparoscopic procedures. In the present analysis, the mean reimbursement for reintervention after inguinal hernia repair was 2568.89 RON (approximately 514 EUR). Laparoscopic reinterventions were associated with higher average reimbursement compared with open procedures (2992.67 RON vs. 2508.35 RON), corresponding to a difference of approximately 484 RON (+19%), with the highest mean reimbursement observed for laparoscopic reinterventions performed in public hospitals (3063.51 RON). This reimbursement model discourages the wider adoption of laparoscopy in the public sector by shifting the additional costs of specialized equipment and consumables to hospitals. Although reimbursement figures do not reflect true hospital costs, they provide an orientative comparison of the financial burden associated with laparoscopic versus open reintervention. In contrast, several Western European countries apply differentiated reimbursement schemes for minimally invasive procedures, using higher tariffs or procedure-specific payments that better account for increased resource utilization and facilitate broader implementation of laparoscopic techniques [33,34,35]. From a health policy perspective, aligning reimbursement mechanisms with procedural complexity and resource utilization may be essential to support equitable access to minimally invasive hernia surgery and to reduce intersectoral disparities in care delivery.

From an international standpoint, Romania demonstrates a noteworthy uptake of laparoscopic inguinal hernia repair despite persistent structural and financial constraints, suggesting that focused training initiatives and surgeon engagement can facilitate the adoption of minimally invasive techniques even in resource-limited healthcare systems. To further advance toward a more balanced and sustainable surgical practice, several policy measures merit consideration. First, reimbursement frameworks should be revised to better reflect the higher resource requirements of laparoscopic surgery, either through adjusted DRG tariffs for minimally invasive procedures or through dedicated funding for laparoscopic equipment and consumables. Second, the establishment of a national hernia registry, modeled on successful Northern European initiatives, would enable systematic collection of long-term outcome data and support continuous quality improvement. Finally, expansion of structured training programs is needed to ensure that laparoscopic expertise is disseminated beyond major academic centers to regional hospitals, where a substantial proportion of the population receives surgical care.

The establishment of a national hernia registry represents a feasible and pragmatic next step for improving quality assessment and equity of care in Romania. At a minimum, such a registry would require a core dataset including patient demographics, hernia type and laterality, surgical approach, mesh use, surgeon and hospital volume, perioperative complications, and reintervention for recurrence. Governance could be coordinated at a national level through professional surgical societies in collaboration with the National Health Insurance House, leveraging existing DRG infrastructure for case identification while supplementing it with prospectively collected clinical variables. Given Romania’s centralized health insurance system and mandatory national reporting of surgical activity, implementation of a focused hernia registry would be technically feasible and could be progressively expanded from high-volume academic centers to regional hospitals.

## 5. Limitations

The inclusion of femoral hernia procedures at the index stage, combined with inguinal-specific recurrence coding during follow-up, reflects inherent limitations of administrative DRG data. Although femoral repairs were infrequent and did not contribute to recurrence events, this approach may marginally affect denominator composition and should be considered when interpreting recurrence estimates.

While non-parametric methods were used for individual-level length of stay (LOS) comparisons due to non-normal data distributions, exploratory analyses comparing pandemic (2020–2021) and post-pandemic (2022–2023) periods relied on aggregated annual mean LOS values. In this context, robust parametric testing was applied, and interpretation focused primarily on effect size magnitude and clinical relevance rather than on statistical significance alone. Hospital-level clustering and the use of multilevel or random-effects models were not feasible in this study, as the anonymized DRG dataset does not allow identification or linkage of individual hospitals across admissions. Given that surgical practice patterns, access to laparoscopic expertise, and referral behavior are strongly center-dependent, unmeasured institutional factors may contribute to residual confounding. Consequently, all regression findings should be interpreted as associative rather than causal.

Detailed operative variables, including the specific surgical technique employed (such as the use of mesh, mesh type, fixation method, and technical nuances of open or laparoscopic repair), as well as surgeon-related factors, are not captured in the national DRG database and therefore could not be included in the analysis. These procedure-specific and operator-dependent characteristics are known to influence both the risk of recurrence and the associated economic burden of hernia surgery. Consequently, the present findings should be interpreted in the context of these data limitations and are intended to describe population-level and system-level patterns of care rather than to allow procedure-specific or surgeon-level comparisons.

The use of reintervention as a proxy for recurrence likely underestimates the true incidence of inguinal hernia recurrence, as a proportion of recurrences are managed conservatively or are not reoperated. This limitation is inherent to administrative datasets and results in systematic under-ascertainment of clinically relevant but untreated recurrences. Consequently, recurrence estimates based on reintervention should be interpreted as minimum estimates rather than true population rates. Similar reintervention-based proxies for recurrence have been widely used in population-based studies relying on administrative data, where direct clinical ascertainment of recurrence is not feasible. Because recurrence was inferred from reintervention, the true clinical recurrence rate is likely underestimated. Patients with clinically evident recurrence who were managed conservatively, declined reintervention, or were not re-admitted to hospital are not captured in administrative DRG data. Consequently, both estimates reported in this study should be interpreted as lower and upper administrative boundaries rather than as true clinical recurrence rates.

## 6. Conclusions

This nationwide five-year analysis suggests that the estimated recurrence rate after inguinal hernia repair in Romania most plausibly ranges between 0.58% and 4.88%, reflecting the limitations of administrative data and the absence of a national prospective hernia registry. The predominance of early reinterventions following the index procedure indicates that technical factors remain a major determinant of recurrence.

Marked differences persist between public and private hospitals, with public institutions managing the majority of inguinal hernia cases and disproportionately absorbing recurrent and clinically complex patients, thereby functioning as a national safety net. Private hospitals, by contrast, operate predominantly within short-stay care pathways. Collectively, these findings indicate that disparities between sectors are not confined to hospitalization duration or economic efficiency but reflect broader structural differences in patient populations, referral dynamics, geographic accessibility to laparoscopic surgery, and institutional capacity.

Although Romania has made meaningful progress in the adoption of laparoscopic inguinal hernia repair, further improvement will require coordinated policy measures aimed at improving equitable access to minimally invasive surgery, strengthening referral pathways, and supporting public hospitals in their central role in managing recurrent and complex inguinal hernia cases. Reimbursement reform, the establishment of a national hernia registry, and the expansion of laparoscopic training beyond academic centers are essential steps to reduce intersectoral disparities and ensure equitable access to high-quality hernia care nationwide.

## Figures and Tables

**Table 1:** Migration Between Private and Public Hospitals at Reintervention.

Initial Intervention	Reintervention Private Hospital	Reintervention Public Hospital	Total
Private Hospital	18	6	24
Public Hospital	20	884	864
Total	38	850	888

**Table 2:** Association Between Comorbidities and Risk of Hernia Recurrence.

Comorbidity	Inguinal Hernia Patients	Recurrence Patients	*p* (<0.05)
**Hypertension**	6218	315	0.4097
**Obesity**	1393	55	0.0920
**Diabetes mellitus**	996	29	0.0030
**Benign prostatic hyperplasia**	1422	100	0.0001
**Heart failure**	919	40	0.4437
**Urinary tract infection**	101	3	0.3711
**Ischemic heart disease**	2395	123	0.5383
**Atrial fibrillation**	888	43	0.9539
**Anemia**	250	18	0.0870
**Chronic kidney disease**	267	17	0.2570

**Table 3:** Average CNAS Reimbursement for Inguinal Hernia Reinterventions.

Reinterventions	Average CNAS (RON)	Average CNAS (EUR)
**Open**	2508.35	513.99
Private Hospitals	2353.90	482.36
Public Hospitals	2513.48	515.06
**Laparoscopic**	2992.67	613.69
Private Hospitals	2458.63	503.82
Public Hospitals	3063.51	628.18

## Data Availability

Data sharing is not applicable to this article.
